# Habitability, Resilience, and Satisfaction in Mexican Homes to COVID-19 Pandemic

**DOI:** 10.3390/ijerph18136993

**Published:** 2021-06-30

**Authors:** Maribel Jaimes Torres, Mónica Aguilera Portillo, Teresa Cuerdo-Vilches, Ignacio Oteiza, Miguel Ángel Navas-Martín

**Affiliations:** 1 Facultad Mexicana de Arquitectura, Diseño y Comunicación, Universidad La Salle Mexico, Ciudad de Mexico 06140, Mexico; m.jaimest@lasallistas.org.mx; 2 Programa de Maestría y Doctorado en Arquitectura, Universidad Nacional Autónoma de Mexico, Ciudad Universitaria, Ciudad de Mexico 04510, Mexico; 3 Instituto de Ciencias de la Construcción Eduardo Torroja, Consejo Superior de Investigaciones Científicas (IETcc-CSIC), 28033 Madrid, Spain; ioteiza@ietcc.csic.es; 4 Escuela Nacional de Sanidad, Instituto de Salud Carlos III (ISCIII), 28029 Madrid, Spain; manavas@isciii.es

**Keywords:** COVID-19 lockdown, home office, telework, households, housing design, mixed-method, habitability, comfort, energy consumption, resilience

## Abstract

Following the 2020 confinement due to the COVID-19 pandemic, housing has become the only safe place and this has exposed inequity in habitability. This research on the reality of confined households and the perception of their homes in the Mexican republic is based on a mixed participatory study, combining quantitative and qualitative approaches. The online questionnaire consisted of 58 questions in the quantitative approximation. The qualitative part required the provision of an image of the workspace, with testimonies and personal reflections. During the lockdown, all participants saw an increase in overall energy consumption; more than half reported not being in thermal comfort; and a third declared deficiencies in noise insulation. Regarding the perception of the telework/tele-study space, we found the following categories: bedrooms, living/dining rooms, studies and others. In addition, respondents had often adapted the workspace for both individual and shared use. In general, the households were satisfied with the size of their houses but would like landscaped spaces or better views outside. Confinement made housing the protective element against the pandemic. The consequences will have an effect globally, so new architectural design paradigms need to be rethought.

## 1. Introduction

The COVID-19 pandemic produced by SARS-CoV-2 has become the highest-mortality respiratory disease since influenza in 1918, which infected 500 million and claimed the lives of 50 million people worldwide [1,2].

In Mexico, the COVID-19 index case was detected on 27 February 2020 [3]. By 11 March, the World Health Organization declared the disease caused by SARSCoV-2 as a *pandemic* [4]. The first two deaths in Mexico would be documented in the Mexico City Civil Registry (CDMX) on 18 March: two men, one in the State of Mexico and one in the CDMX, 41 and 80 years old, respectively [5,6]. On 30 March, in the Official Journal of the Federation, the health emergency of the epidemic was decreed [7], caused by the SARS-CoV-2, due to the increase in cases and deaths caused by COVID-19 [3], which, up to that point, amounted to 1094 confirmed cases and 28 deaths [8].

Without clinical treatment for cure or prevention against COVID-19, in March 2020, preventive measures of non-pharmaceutical contagion, mainly hand washing, covering up when coughing or sneezing, avoiding physical contact in greetings, and maintaining physical distance [9,10], became the strategy to be followed globally, as the most effective way known so far to prevent the contagions of the new coronavirus was physical distancing [11].

The *Healthy Distance National Campaign* lasted from 23 March to 30 May 2020 [3]; where the suspension of all non-essential activities was mandatory [10]. Later on, the Government of Mexico opted for voluntary confinement, mainly due to the social situation of poverty in the country, and the necessity that Mexicans went out to work every day in order to survive. This campaign was named *New Normality* and has run from 31 May until now. This situation is dictated through the epidemiological traffic light, which is a tool that measures the mortality rate, hospital capacity and new infections, in order to grant permission for non-essential activities, such as the return to face-to-face activities. Health promotion policies contemplated measures to try to curb contagion by controlling the flows of bars and commercial squares, in collective transport, the indefinite closure of schools, parks and gymnasiums [12].

The population was never necessarily locked up. Instead, the government made the population aware of the responsibility of each citizen to not use public spaces and to shelter at home. Citizens were made to understand that the only place to decrease the chance of contagion was the house. *Stay home* [13] became the voluntary confinement strategy that has lasted about thirteen months, with its critical phases corresponding to the red light imposed on the CDMX in May and December 2020, and January and February 2021. At the end of September 2020, Mexico finished seventh in confirmed cases, and fourth place in deaths worldwide, making it one of the countries most affected by the effects of the pandemic [14]. Exceeding any estimate, and despite these above strategies, the figures as of 31 December 2020 in the Mexican Republic report 1, 426,094 confirmed cases and 125,807 deaths in national territory [15].

Houses sheltered both the sick and the healthy, and this reoriented the habitability in the house. Given the uncertainty of the duration of the COVID-19 pandemic, and physical distancing measures, the response of the world’s population has tended to digital migration, and although in this same decade there had been situations of confinement in Mexico, telework and tele-study were never experienced as they were in 2020.

In 2009, in Mexico, the first pandemic of the 21st century due to influenza A virus (H1N1) [16] caused the population to be in voluntary confinement for about a month. With a less cyber-connected population, telework and tele-study were not detonated widely as in the COVID-19 pandemic. Even two years ago (2017), after the September earthquakes in southeastern Mexico and the country’s capital, during the suspension of academic activities by the emergency declaration, there was a widespread response to absorbing work at home.

At this time, the perfect elements were combined to detonate a new era for housing, with its new predominant activities, telework and tele-study. Technological advancement and unprecedented physical distancing forced companies, schools, and any businesses into the digital medium. During the pandemic, the house was not only the place of shelter from the virus, but there was a possibility of changing the way households were inhabited and temporarily transforming them into classrooms and offices. The house became the vital core of the new era, which is a new feature for the design of the architectural program of the house and the city of the future.

The public health threat posed by COVID-19 exposes this other latent one related to the habitability of our homes. Since we have been overexposed for more than 10 months to a single enclosure, the vulnerability of homes for a large number of dwellings is revealed, especially in the peripheral neighborhoods of the cities, where homes have extremely small dimensions, are overcrowded and do not have basic services such as electricity, water or the internet.

Numerous studies have been conducted in order to understand the psychological effect of indoor and outdoor spaces on human health. Highlights include those that focus on indoor spaces, since even before confinement it was seen that, on average, people spend 90% of their time indoors [17]. It is known that the conditions of habitability in the homes go hand in hand with the socioeconomic status or social class of their occupants. In this regard, the World Health Organization (WHO) notes that there is also a direct link between life quality and expectancy, and housing living conditions [18]. The literature also establishes studies in which young students have moderate to severe degrees of depression, and the relationship that exists with the small dimensions [19]. These facts have not gone unnoticed in these moments of confinement.

Space design issues, such as the number of windows, the number of m^2^ per occupant, and the distribution of spaces, have been a determining factor for contagion among the inhabitants of the same house, and have affected their capacities to telework and tele-study during this confinement period [20,21].

Among the literature, one article stands out [22], on which this research is based, which consisted of a mixed-method study, applying two online questionnaires to 1800 people in Spain, to analyze the resilience of homes during the COVID-19 confinement. It distinguished the presence of outdoor spaces in the houses, such as terraces, as having a direct and positive impact on the natural lighting and air quality inside the houses.

Yet another example of the importance of the habitability conditions during the confinement was reported in an study carried out in Culiacán, México [23]. In this research, which was also performed by an online questionnaire, 281 respondents who live in one of the states more affected in Mexico were analyzed. The main objective of this investigation was also to understand how social distancing had affected the way that population lived. In this study, Verdugo [23] defined that the design of spaces in the house determines the permanence of its inhabitants within its homes. In addition, this research reported that only 4 months after isolation was established, reports of domestic violence increased dramatically, which invites reflection regarding whether the houses were prepared for long periods of confinement and whether it was possible for them to take on the infrastructure of the house, the office, and the school, all at the same time.

The National Autonomous University of Mexico also conducted an original investigation in May 2020 in Mexico City [24]. This study was also carried out through an online questionnaire answered by 5000 people. It was also intended to collect information on the living conditions and the changes perceived by the inhabitants during the period of confinement. In it, the researchers point out that confinement measures amplify social, economic and gender inequalities. Therefore, they recognize that it is important to understand whether the home offers adequate living conditions. They concluded in this preliminary observation that one of the main problems encountered during this confinement, especially in popular colonies, was the overcrowding in homes due to the number of inhabitants and the size of the housing.

Finally, another study carried out in Tijuana, Mexico, which was also based on an online questionnaire, concludes that mostly the negative effects of social isolation affect the living conditions of those with small homes and who are found in poor neighborhoods [25].

With all of the above, a knowledge gap is detected within the literature, such that it presented a full picture of people’s home perceptions, involving materials, distribution of living spaces, habitability and comfort, as well as aspects of the quality of lighting and ventilation. It is necessary, then, to portray how housing has responded to this dynamic situation.

The importance of studying situations of the extended use of housing lies in understanding the needs, preferences and deficiencies of houses in order to improve the living conditions of housing in developing countries, in environments with a lack of services and in small houses. Unfortunately, social distancing scenarios can be repeated in the post-COVID-19 era, due to high temperatures or extreme pollution. Moreover, people with motor or mental disabilities, and elderly people, live in this condition of housing overexposure all the time, and their quality of life is partly determined by their homes.

## 2. Theoretical Foundation

People actively interact with the buildings and built environments they inhabit. The behavior of people occupying indoor spaces is essential for their proper functioning and performance during their lifetime, as well as for their maintenance. In some respects, people also intervene decisively in aspects such as the efficient use of resources, including energy and indoor environmental quality. Therefore, knowing the needs of these people, their use patterns as well as their space perception, is vital in knowing whether the building or living space (domestic or not), is satisfactory for the performance of their tasks, their well-being, and even for their own health.

There is no theory that comprehensively defines housing, due to its nature of a complex phenomenon that must be approached from different points of view [26]. However, according to Anita Blessing, “Housing’s role in the welfare state has been famously described as awkward and uncertain” [27]. Now, more than ever, the relationship between housing and mental health is relevant, especially in this period of prolonged confinement. Due to the fact that confinement can have negative effects on people’s mental health, which may make people more susceptible to experiencing periods of anxiety, insomnia, anger, irritability, stress, this reveals the importance of built environments [19].

Studies indicate that, in Mexico, the loss of the daily routine, the fear of being infected, and discussions and conflicts with the family, are negative factors perceived by the population that determine teleworking. Therefore, Villavicencio-Ayub proposes after a study carried out during the months of confinement, that “*the higher productivity and satisfaction with work at home, the less disruption of daily life*”. The spaces for preserving daily life are relevant to preserving, at the same time, family life and the mental health of the household members [28].

Teleworking allows workers to carry out work activities in different places, one of them being the home. Through information technologies, teleworkers can access their work activity from anywhere [29]. Technological development has contributed to various forms of remote work, in addition to making it accessible to more people [30].

Several authors highlight the (theoretical) benefits that telework brings to people who work remotely. The most important factors turned out to be flexibility, greater autonomy and having more time to spend with the family or for leisure [29]. These benefits have an impact on the health and well-being of workers, taking into account that flexibility contributes to reducing stress, in addition to favoring family conciliation [31].

Workplace conditions job performance. An inappropriate workplace negatively influences worker’s productivity. Instead, a suitable location contributes to higher accomplishments [29]. It is the same with the environment that surrounds these workspaces. Not having exclusive spaces for this activity can generate family conflicts. This leads to having to share spaces designed for other purposes, such as places for conversation or relaxation [32].

The conditions of teleworking in a normal situation, or even from the theoretical perspective with respect to the conditions during the pandemic, have been very different. Teleworking, in principle, facilitates family conciliation, gives the workers greater flexibility and allows them more free time, as I have seen previously. However, working during the COVID-19 pandemic has not followed the usual teleworking pattern. This is due to the extreme confinement, the anxiety caused by the health emergency situation itself and the economic crisis situation. It has caused greater psychosocial and ergonomic risks in the health of teleworkers [33]. According to several studies, a greater increase in post-traumatic stress disorders was associated with confinement due to the pandemic [34]. Some people were negatively impacted by working at home instead of their workplace, causing them more stress [34]. Among the various reasons, some authors point out that stress may be caused by the presence of children during the working day, as schools are closed. Likewise, teleworking can cause teleworkers to dedicate more hours to work because they do not know how to differentiate the boundaries between private and work life [35]. Although some studies found positive impacts, some teleworkers highlighted that during confinement, working from home allowed them to be more efficient and they also experienced less exhaustion [34].

According to the survey “Measurement of the Impact of COVID-19 in Education (ECOVID-ED) 2020” from the National Institute of Statistics and Geography of Mexico, only 38.4% of the population aged between 19 and 24 years old were enrolled in the 2019–2020 school year. In fact, this could be already considered a low percentage in relation to younger populations (for instance, 98.7% of children aged 6 to 12 were enrolled to continue with their studies). For the following academic year, the percentage of inscribed people aged 19 to 24 decreased to 31.6%. Those who did not enroll in the academic year alluded to several causes: COVID-19 (5.2%), economic reasons (12.5%), work duties (13.1%), and others (37.7%) [36].

A specific piece of research related to Mexican university students, based on the General Health Questionnaire (GHQ-28) and other scales, evidenced psychological effects of COVID-19 pandemic on students. Among them, sleep problems, stress and social dysfunction stood out, due to prolonged confinement and intensive work hours. Once again, the environment played an important role in mental health, since factors such as natural lighting and ventilation conditioned habitability. It was necessary to spatially understand the domestic milieu, subjected to an intensive use and habit changes due to teleworking and tele-studying [37].

This is the reason why specific and heterogeneous problems regarding housing could not be susceptible to a single unified theory, and even more so, given this extreme and novel context.

In the residential sector, COVID-19 confinement has led to an alteration in temporary settings and habits in the homes, including teleworking or tele-study as an incipient modality in many countries. It has had consequences for the use of indoor spaces, energy consumption, and perceived indoor environmental quality, including aspects of thermal, light and acoustic comfort, and air quality.

To determine this household-dwelling interaction, the most commonly used techniques are surveys or questionnaires, where aspects of habitability and comfort, availability and use of the specific space, system, or device (for example, to evaluate energy or indoor environmental issues) are requested. This information is often asked to case-study households, distinguishing them by cohabitant(s), day/night patterns, weekdays/weekends [38,39], and even hourly-usage logging. These questionnaires or surveys are usually carried out in person, with a visit to the domestic spaces to obtain design or technical data to assist in the evaluation. A context narrative usually accompanies all mentioned, to contrast data from other objective techniques, such as the monitoring of energy or environmental parameters, or predictive computer simulation [40].

Therefore, even if some studies do not use this designation [41], they are based on mixed research methods. Such methods are justified on the use of quantitative and qualitative components throughout the research. The interrelationship of both levels of approximation for greater complementarity [42] is characteristic in them.

Thus, these mixed approaches use the two approaches to quantify certain indicators, while qualitative information provides details and nuances that enrich and assist in the interpretation of the object of study, and the phenomenon itself [43,44,45,46], validating the results obtained by triangulation [47]. However, these approaches are usually applied with reduced samples, for non-probabilistic studies, and non-generalized results [48].

The COVID-19 pandemic situation has limited research tools. This led scientists to use qualitative and quantitative research to better complement their studies [49]. There are several studies that have used both perspectives during the pandemic, usually utilizing questionnaires for quantitative data and interviews or open questions for qualitative ones [50,51,52].

Including open questions in questionnaires allows aspects not previously considered or other insights for the researcher to be covered, which are typical of exploratory studies in the face of little-known phenomena, or those new to society [47,53]. This resource has also been widely used in self-completed online forms [51,53]. However, other mechanisms for obtaining information, or the use of different tools such as photographs, have not been much explored. This data could be obtained by adapting visual and participatory research techniques to the context of pandemic [54]. These, although little used in the technical field of analysis of the built environment, could be remarkably interesting for covering the domestic spatial analysis [22].

Other studies have used samples already selected in cohort or longitudinal studies to make comparative analyses [55,56,57]. This option, although desirable, is not common in the field of building, construction and internal space design, due to the lack of access to relevant samples.

During this period, studies abound aimed at delving into the health and welfare implications of people’s physical, psychological, and emotional well-being [58,59].

Some more targeted studies on aggregate energy use in the residential sector have been based on secondary data provided by suppliers and distributors of energy sources to housing [60]. However, from the point of view from inside homes, no contributions have been found that specify how these habits have been altered in housing, and the consequences the lockdown has had in terms of spatial and behavioral adaptations, in the availability and use of energy, the perception of comfort (since housing has now been permanently occupied), and the satisfaction generated [22]. These studies are even more relevant and necessary for developing countries.

With this contribution, the authors seek to promote the knowledge regarding the performance of life inside the homes, and the capacity to adapt to the new needs of both the households and the dwellings themselves. To this end, the analysis is based on the main indicators or variables related to the occupation and use patterns, which are habitability, comfort, indoor environmental quality, and the satisfaction degree of Mexican homes. It also includes qualitative analysis around telework/tele-study spaces, as well as narratives on other references and suggestions from participants who voluntarily wanted to add them in their questionnaires.

Moreover, this study hopes to contribute to the knowledge of the confinement phenomenon in the homes, what implications it has had for households, and what has been required for dwellings in terms of space and resources for the adaptation to this public health measure. Finally, this approach is intended to encourage discourse aimed at responding to population needs in terms of social, energy, environmental, economic and health impacts associated with overexposure to the domestic domain, whatever the physical and spatial capacities. These responses can be materialized in a project, with technical solutions, as well as mitigation or preventive policies in the face of possible similar future scenarios, with the development of appropriate contingency strategies and plans, which favor the most vulnerable, who have suffered even more from the havoc of COVID-19.

## 3. Materials and Methods

### 3.1. Hypothesis, Objectives, Research Questions and Methods

The starting hypothesis is that the confinement of households has led to a change in habits, use of spaces, and adaptation to circumstances (resilience), giving rise to alterations in energy consumption, as well as in the perception of housing in general and in its preferences. The objective therefore focuses on assessing the use, perception and degree of satisfaction of the Mexican population regarding the habitability of their homes during confinement. As specific objectives of the complete study, it seeks to: (1) delve into the issues of design, performance and the availability and use of the different house services, to better understand how confinement has been experienced; and (2) understand the reflections and interventions that this extreme situation can generate in the design and rehabilitation of Mexican homes, as well as in their energy and environmental implications, and finally the repercussions on the health of their inhabitants. In turn, the research questions were aimed at exposing aspects not covered by the questionnaire: Q1: has it generated thoughts regarding the popular experience around confinement and housing, any reflection or suggestion they want to share with their peers? Q2: if the participant has teleworked or tele-studied, in which spatial environment has he performed that task, and how has the experience been of this telework space?

To carry out this research, the study on social confinement (COVID-19), housing and habitability developed in Spain, was used as a reference [22]. The main objective of the Spanish study was to find out the perception of the population about confinement, its relationship with housing, and the degree of resilience to the extreme circumstances caused by the pandemic.

For the development of the study in Mexico, the same methodology of the reference counterpart was used, this being a mixed study based on quantitative and qualitative approaches. For its implementation, the cultural and linguistic differences of both countries were taken into account, in addition to the redesign of some questions, in order to adapt to the specific objectives for the analysis of the context of Mexican society.

### 3.2. Data Collection and Recruitment

The study was based on non-probability sampling. Due to time and resource constraints, the opportunity to conduct research [47] could not be wasted, given the context of people in confined situation. To do this, two forms of sampling were used, convenience and snowball [47,53].

This technique of data collection for convenience allows more people to be reached, because anyone can participate, as it is recommended for exploratory studies. One of the most common ways to apply it is through online questionnaires [53].

Snowball sampling allows the selection of cases in a given network; taking advantage of participants’ interrelationships to reach others [53]. It is often used, in fact, in hard-to-reach or safety emergencies, as it is the most recommended option for accessing subjects of study [61].

As for the origin of the exhibition among students of Architecture, the students were used as more initial and active participants, who also promoted the dissemination of the questionnaire, since the initiative of the study arose from two researchers and professors of the La Salle Mexico and National Autonomous of Mexico Universities (UNAM).

Different channels of dissemination were used to achieve the greatest possible participation, considering the limitations of the pandemic situation. These media were mainly by email, institutional web, social media and instant messaging apps (such as *Whatsapp*^®^. In addition, using students would allow their opinion to be taken in to account for future analysis oriented to a vision from architectural design. In a more detailed study, this could be disintegrated from the opinion of participants unqualified in the Architecture field, through a specific question with a dichotomic response (yes/no), to distinguish, in that case, the sample according to its training in that area.

Regarding ethical issues, researchers followed the Declaration of Helsinki as well as the recommendations given in the Spanish eponymous study, and their corresponding Ethics Committee’s approval. On the other hand, all participants gave their consent before accessing the questionnaire, through an Ethical Statement, in which they were previously informed of the study objective and the research team who launched it. It was also mandatory to participate, to declare: (1) to be over 18 years old; (2) to occupy the main dwelling during confinement; (3) to be the only representative of the household participating in the study; and (4) to participate freely and voluntarily all the time. They were also informed that the questionnaire was completely anonymous. For the inclusion of photographs, it was reported that these should not include nor show any trait, element, part or object that could involve the identification of persons either directly or indirectly, in accordance with current legislation on data protection.

### 3.3. The Survey (Quantitative Approach)

For data collection, it was established with an anonymous mixed questionnaire online, through the *SurveyMonkey platform*^®^. This questionnaire consisted of 58 questions with quantitative approximation and 4 with a qualitative approach, with an average response time of about 15–20 min. The questions fall into seven categories. The categories in the first part dealt with: sociodemographic data (including specific training in Architecture); characteristics of the dwellings; habitability; perceived comfort; habits of use and occupation (including special mention of the characteristics of telework); energy equipment and services in the house) and energy consumption patterns.

The quantified variables for each category are specified in Table 1.

Given the variables collected for this study, cited in Table 1, three of them were selected to represent and analyze the following aspects: home performance, behavior and adaptation, according to house features:

- Performance: the selected variable was “temporary dedication to tasks”. The original question was based on a Likert scale 1–5, for any of the tasks given; being 1 being less time-dedication, and 5, most time-dedication. The task chosen was “telework/study”, to determine if general housing features affected to those people who were devoted to telework, or not.
- Behavior: the variable chosen was “modified habits”. The original multiple-choice question was: “Indicate, of the following habits, which have been altered during confinement”. Fourteen potential responses were given.
- Adaptation: the variable defining this concept was “house spatial adaptation”. The original question was “Did you have to adapt your dwelling of some of its spaces for any of these causes?”. It was a multiple-choice question, and there were six potential responses.

The cross-tabs and relationships stablished around these three variables are presented in Section 4.9.

### 3.4. Photos and Testimonies (Qualitative Approach)

As for the second part of the study, included in the same form, it was addressed from a qualitative perspective. Through the same data collection platform, participants were asked to take an image defining the remote work/study space they had at home. They were also urged to tag the photo taken with three keywords, as well as to explain the most noteworthy features of the photo they had taken and the subject. Finally, participants were encouraged to share a suggestion or reflection related to confinement and housing. To this end, an adaptation of the methodology based on the use of images was made [22,62] and it was implemented in the current context of pandemic [54].

### 3.5. Data Analysis

#### 3.5.1. Quantitative Data Analysis

A descriptive statistical analysis was performed for quantitative data. For this, the wrong records were deleted, and some variables were recoded for better interpretation of the data. For analysis, the frequencies and percentages of the different variables were calculated using IBM SPSS version 26 statistical software.

#### 3.5.2. Qualitative Data Analysis

For qualitative data, the NVIVO release 1.3.1 qualitative analysis program was used to code and categorize the images, as well as to search for frequencies and word clouds of the participants’ tags and for the two open questions. In addition, the most significant verbatim records linked to the participants’ testimonies were selected. For the creation of term frequency tables, and word clouds, word frequency search was performed with the criterion of having a minimum length of 3 letters, matching derived words. Empty words (meaningless) were excluded.

## 4. Results

The information provided by the participants was obtained between 7 August and 30 October 2020, a temporary period in which the Mexican population was in confinement. A total of 1008 responses were received, with 970 valid for the quantitative part. With respect to the qualitative part, 558 valid responses were obtained, and the subsequent images, 1599 labels, 539 explanatory responses relating to images, and 460 responses on reflections and suggestions related to housing and confinement.

The distribution of the sample obtained according to the contact channel was: 18.2% by mail, 10% using institutional web, 56.5% through *Whatsapp^®^*, 13% by social networks, and 2.3% by media.

Results obtained through the mixed questionnaire are presented in next sections.

### 4.1. Sociodemographic Profile of Participants

Below, the descriptive statistics summary is given for the total sample, according to the main sociodemographic variables (Table 2).

**Figure 1:**
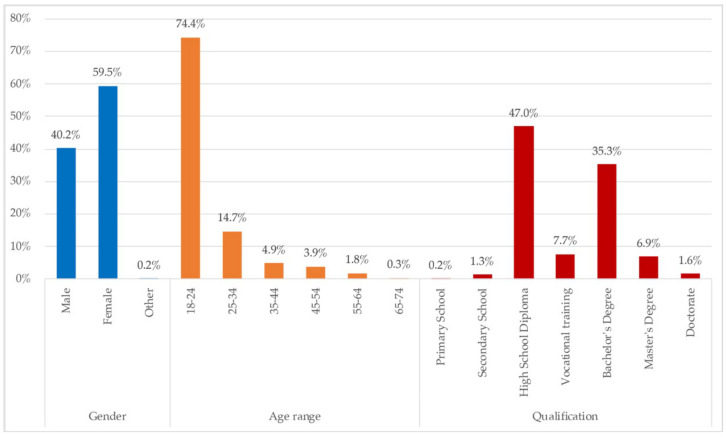
Sociodemographic results of participants.

On the sociodemographic distribution shown in Figure 1, it is understood that, because the data collection was carried out mainly among undergraduate and postgraduate university students, there is a large number of the sample of people between 18 and 25 years old, and that their maximum degree of studies is High School Diploma, firstly, followed by the option of Bachelor’s Degree. Gender participation exhibits mostly women; roughly 60%.

### 4.2. General Housing Taxonomy

**Figure 2:**
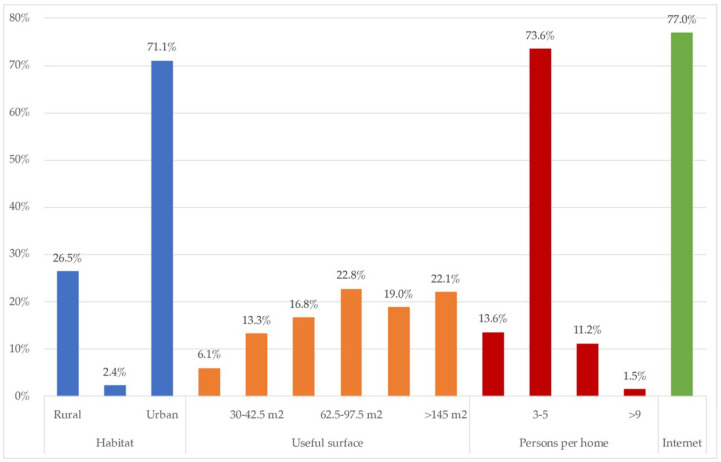
Profile of the households participating in the study.

On the profile of the homes and housing participating in the study, Figure 2 obtained information about habitat distribution, useful housing area, housing types, number of people per household, and type of internet connection.

The sample of participating homes belonged mainly to urban habitats (71.7%). The useful surface area of dwellings was equally distributed for almost all the ranges. By contrast, the main household composition was of three to five household members (73.6%), and 12.8% with six of more, so the useful surface was expected to be at least more represented in the larger surface ranges. Therefore, it is understandable that a part of the respondents lived through the confinement in small houses, according to their cohabitants. A very high percentage of households had a broadband internet connection internet (77%).

**Figure 3:**
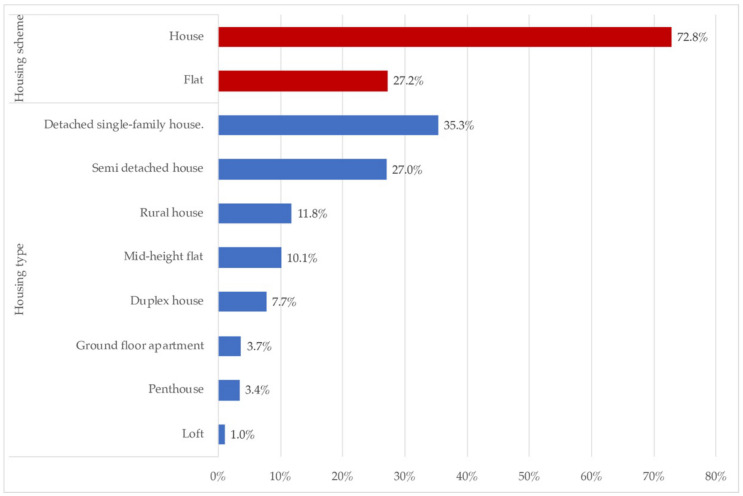
Schemes and housing types of the participating sample.

**Figure 4:**
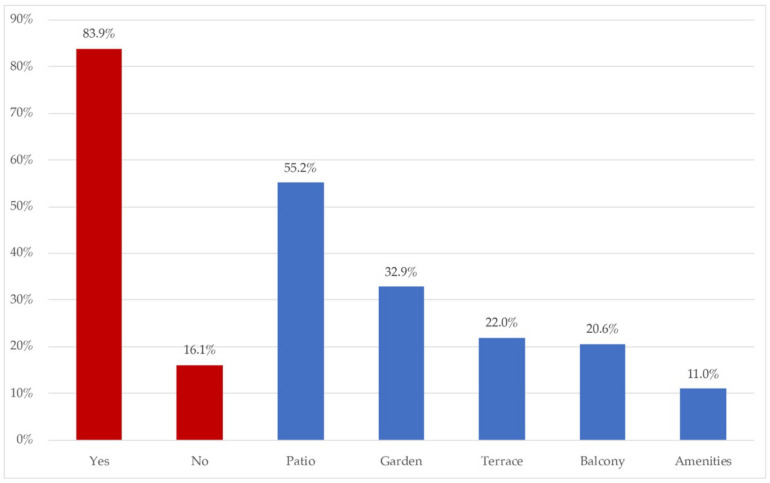
Availability and type of open spaces offered by participating homes.

The following is the data obtained as to the environment in which the houses were located, as well as the availability of spaces in contact with the outside of the houses. Figure 3 presents the distribution for the different types of participating dwellings. Figure 4 sets out the results on the proportion of homes with available outdoor spaces, and the nature of them.

The Figure 3 represents the housing type and scheme among the home participants. Almost three quarters of the sample lived in houses, distributed in detached and semi-detached (62.3%), followed by rural houses (11.8%). The remaining classifications were included as different types of flats (25.9%).

Figure 4 shows the availability of open spaces in surveyed homes, and if available, which kind of open spaces they were. 83.9% of the sample had an open space, mainly patios (55.2%) (due to the fact that most of the sample had houses, not flats), followed by 22% who had gardens. Terraces and balconies represented 44.6% (present both in houses and flats), and finally there was a minority which had access to amenities (11%).

### 4.3. Indoor Environmental Quality

**Figure 5:**
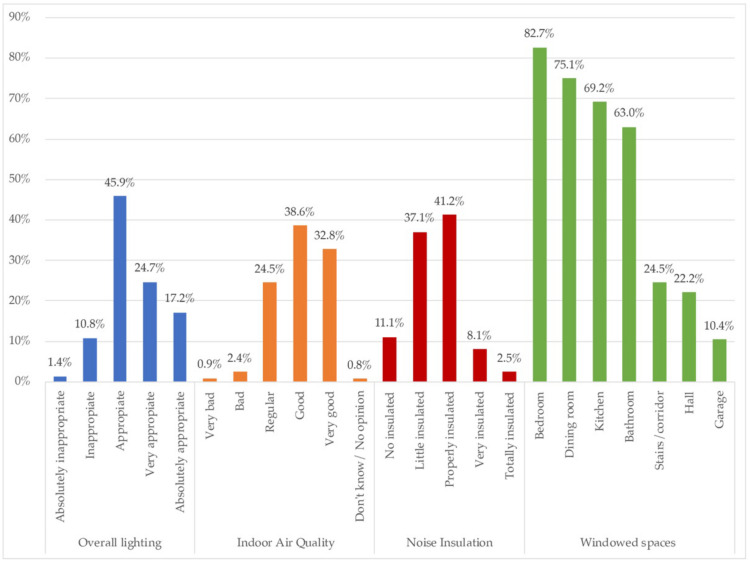
Indoor environment quality (lighting, air quality, noise insulation, and room windows).

Data were collected on how people perceived the quality of the indoor environment, in terms of natural lighting, air quality, noise insulation, and the availability of windows by room type, as shown in Figure 5.

In the Figure 5, aspects of indoor environment quality were represented. On the overall lighting, in general it could be said that the perception is good or very good for 87.7%. 41.9% perceived it very or absolutely appropriate, but 45.9% qualified it as just appropriate. Only 12.2% felt the home lighting inappropriate.

According to the indoor air quality, it was declared good or very good for 71.4%, regular for 24.5% and bad or very bad just for 3.3%. So, similar to the perception of lighting, in general surveyed respondents were satisfied with the air quality. Related to the air quality, the location of windows in the homes was highlighted, with bedrooms being the rooms most likely to have windows (82.7%), and dining rooms (75.1%), followed by kitchens (69.2%) and bathrooms (63%). Those spaces less windowed were stairs/corridors (24.5%), halls (22.2%) and garages (10.4%). Therefore, homes are more windowed where people spend more time, standing out the main rooms, such as bedrooms and dining rooms. Bearing in mind that Mexico has high rates of self-construction, this indicates the preference for the location of windows when they have to choose and take a decission.

Finally, related to noise insulation, respondents declared their homes to have poor (48.2%) or just enough noise insulation (41.2%), with those who declared good noise insulation in their homes being a great minority (10.6%). Almost the half of the sample had poor insulation. This could be partially related to the same reason, the self-construction, and thus, the regulatory non-compliance in terms of protection against noise (also the case of the lack of thermal insulation).

### 4.4. Comfort

**Figure 6:**
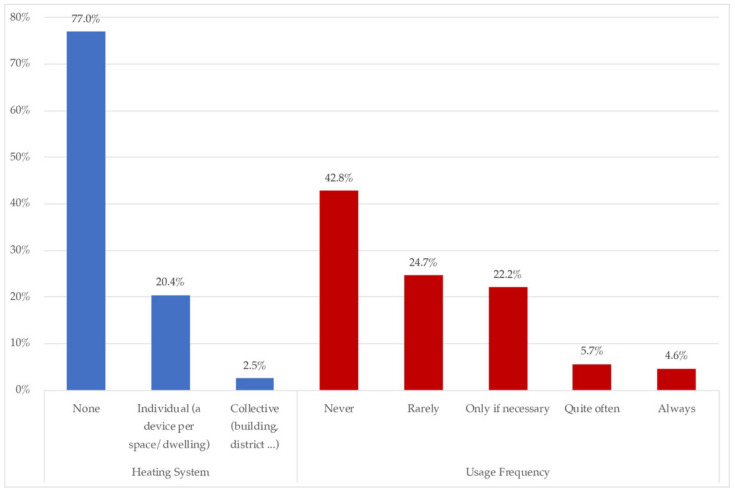
Availability and frequency of use of the heating system.

**Figure 7:**
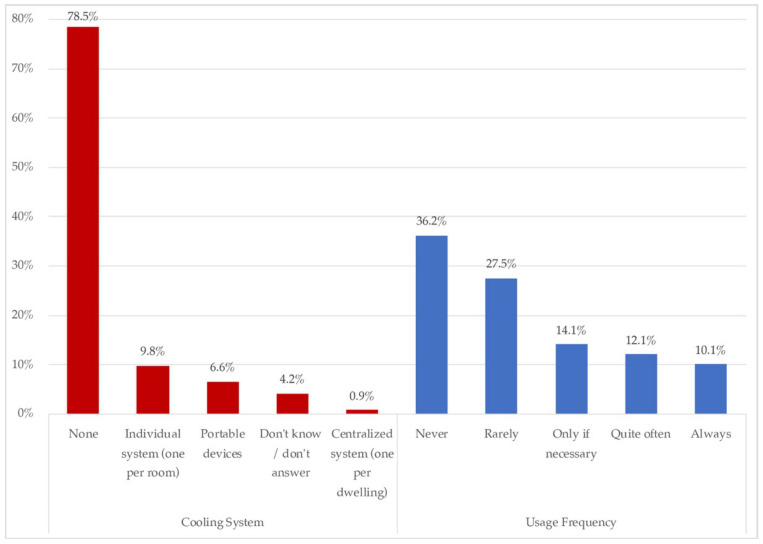
Availability and frequency of use of the cooling system.

In this area, the thermal conditioning of housing, by system, comparative use (regular/confinement), and the perceived comfort (ISO 7730/ASHRAE standard 55) are highlighted. Figure 6 and Figure 7 show the availability and frequency in the use of heating and cooling.

Heating systems were available for 77.1% of the homes surveyed, so for almost a quarter there was no heating system. If available, the use frequency was “never” for 42.8%, and “rarely” for a quarter. 22.2% switched on the heating only if was necessary. The percentage of homes demanding greater heating use was 10.3%.

Figure 7 shows that 78.5% of homes didn’t have any cooling system, whilst 17.3% did have one. Among those 17.3%, almost all of them were individual, either individual per room (9.8%), or portable (6.6%).

The comfort declared by participants, according to the Likert 1–7 Scale set out in ASHRAE standard 55 [63], revealed that 47.78% of households felt comfortable indoors. The remaining 52.22% of respondents were not in comfort. A percentage of 30.69% declared themselves to be uncomfortable because of cold, whilst a 21.54% declared it was due to heat.

### 4.5. Behavioral Patterns (Use and Occupation)

**Figure 8:**
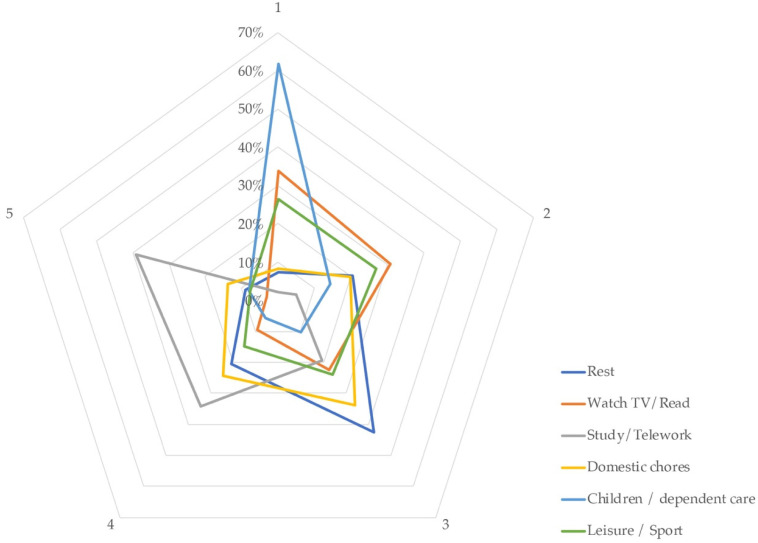
Time-dedication distribution of participants to activities at home, in COVID-19 context.

**Figure 9:**
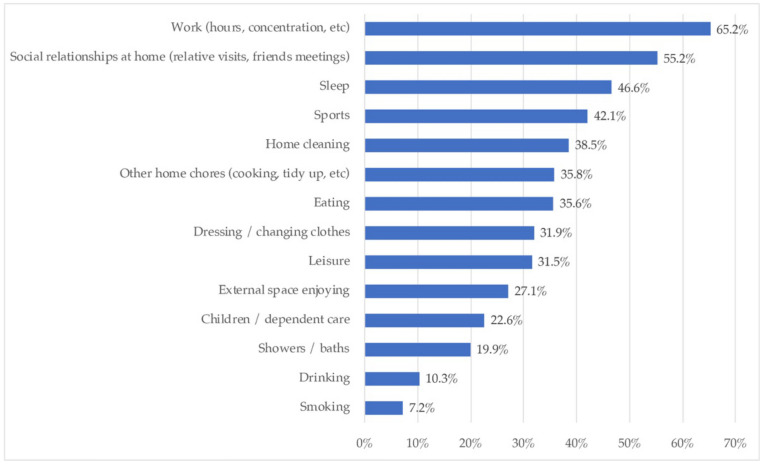
Main household habits that were modified during confinement.

**Figure 10:**
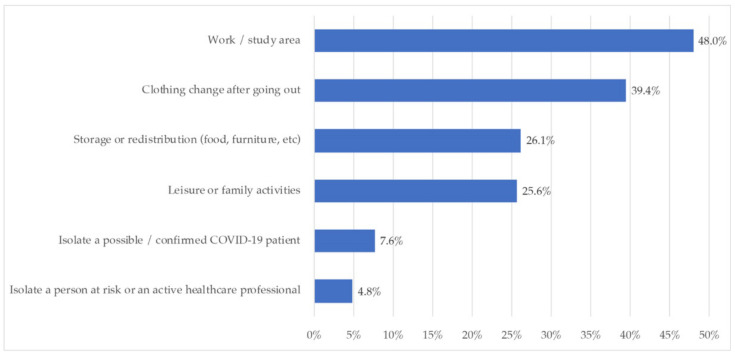
Main adaptations to the dwelling space during the lockdown.

Data were collected on the time-dedication distribution of participants to particular tasks during lockdown (Figure 8), the main habits that were modified in this period (Figure 9), and finally the physical or functional adaptations made to home spaces by their inhabitants (Figure 10). For each task, home representatives voted in a Likert scale from 1 (less time-dedicated) to 5 (most time-dedicated). Six tasks were offered: rest, watch TV/read, study/telework, domestic chores, children/dependent care, and leisure/sport.

Figure 8 shows the polygons generated which accumulated responses, indicating that telework and study are the most time-devoted tasks, followed by domestic chores and rest. After that, people spent their time watching TV or reading, and participating in leisure or sport activities in the same proportion, and finally, they dedicated the least amount of time to children/dependent care.

Figure 9 collected the habits that households changed during the lockdown. They declared to have changed mainly work habits (65.2%), social relations at home (55.2%), sleep (46.1%), and domestic chores: home cleaning (38.5%) and others (35.8%). Eating was also altered for 35.6%, and clothes-changing and leisure changed for almost a third of the respondents. Enjoying external space was perceived to be altered for 27.1% (which is similar to the percentage of flats, which had no patios or gardens). Around a fifth of the sample altered the children/dependent care and showers/baths.

Figure 10 shows the main spatial, physical, or functional adaptations made by participants in their homes during this period.

Regarding the spatial, physical or functional adaptations in the homes, again, those related to the work or study areas were the most common (48.5%), followed by clothing change after going out (39.4%). Storage or redistribution changes were made by 26.1%, and leisure of family activities by 25.6%. Finally, isolation for COVID-19 reasons (7.6%) or cohabitants with risk, including active healthcare personnel of health professionals, meant less spatial adaptations for the households.

### 4.6. Facilities and Appliances

**Figure 11:**
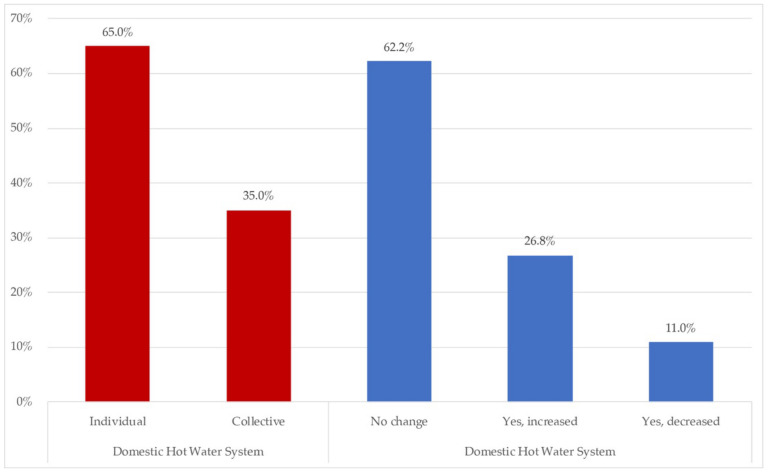
Type of domestic hot water system and perception of its frequency of use during confinement.

**Figure 12:**
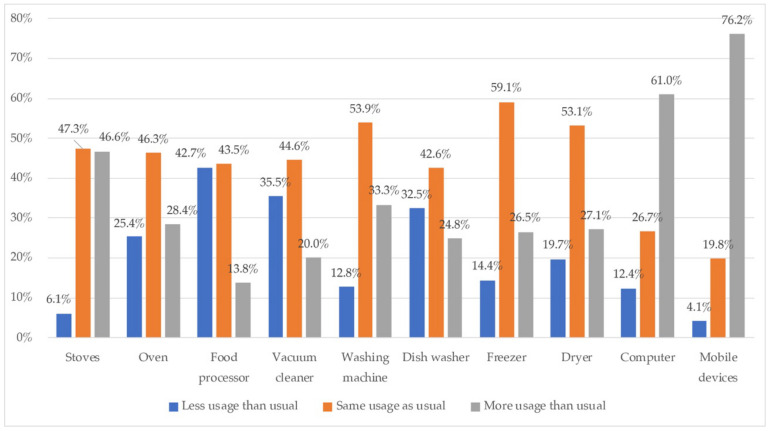
Comparison of frequency of use related to appliances in the home before and during the study period.

This section detailed issues related to the type of energy used for the domestic hot water (DHW) system and its use, compared to normal use (Figure 11) and the intensity of use of household appliances with respect to pre-confinement use (Figure 12).

Figure 11 shows the types of domestic hot water systems and their use with respect to pre-confinement usage.

Related to domestic hot water, 65% of houses had individual systems, whilst 35% were collective. The respondents did not alter the habits for this service in 62.2% of cases, with 26.8% increasing their use. Just 11% decreased the use of hot water during confinement.

Information on the use of electrical and electronic equipment before and during lockdown is reported in Figure 12.

On the compared use of appliances (electrical or electronic) before and during lockdown, technological devices were highlighted by increased usage (mobile devices, 76.2% and computers 61%). Additionally, stove activity also increased in 46.6% of cases, and washing machine use increased for 33.3%, but mainly the use was the same for 53.9%. Other appliances such as freezers, dryers, and stoves, had the same use in this period for almost a half of the sample. None of the appliances showed less usage than usual for most of the sample.

### 4.7. Energy Expenditures and Saving Strategies

In this section, the aspects around the expected change in energy expenditure in the confinement period are highlighted, with respect to the common one for households. It also reflects the possible energy-saving strategies implemented by families during this period.

The perception on change in energy bills showed that 45.19% of participants thought that their bills would change “enough”, understood to mean a change perceptible but not significant enough to create a great alteration on general expenditures. Of the sample, 18.51% suggested a relevant alteration in energy bills during confinement, whilst 36.3% would not perceive a significant change in their energy expenditures.

**Figure 13:**
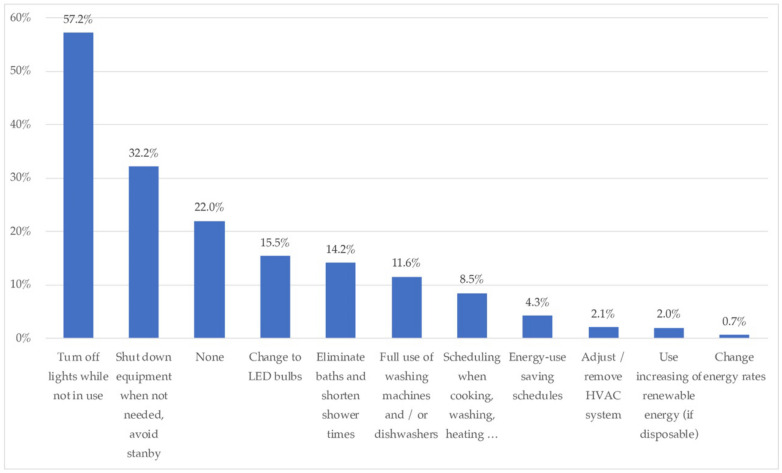
Energy saving strategies carried out in the households participating in the study period.

The domestic energy saving strategies carried out during confinement are shown in Figure 13.

In terms of the energy saving strategies that households took up during the lockdown, turning off lights was the most frequently applied (57.2%), followed by switching off equipment when it was not in use (32.2%). The percentage of respondents that did not apply any saving measures in their homes was 22%. Other, less common measures were the substitution of lightbulbs with LEDs (15.5%), shortening baths and showers (14.3%), and resorting to filling washing machines and dishwashers to be more efficient (11.6%). The remaining measures did not reach 10% application.

### 4.8. Desired Housing Improvements

**Figure 14:**
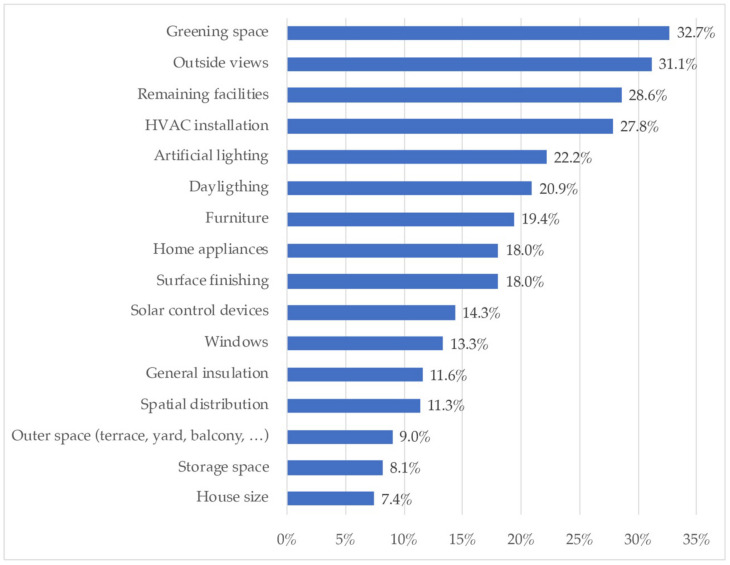
Aspects about the housing that the inhabitants would change, if possible.

Finally, Figure 14 reflects those aspects that participating households would change about their home, if they could.

Figure 14 presents the aspects that respondents would like to change about their homes, if they could. The most popular aspects were green spaces (32.7%), and views to the outside (31.1%), followed by facilities (HVAC, 27.8%, and others, 28.6%), artificial lighting 22.2% and daylight (20.9%). Interior aspects were then highlighted, such as furniture (19.4%), appliances (18%), surface finishing (18%), solar control devices (14.3%), and also aspects related to external surroundings, such as quality of windows (13.3%), insulation (11.6%), and spatial distribution (11.3%). Other aspects with under 10% of responses were outer space, storage, and house size.

### 4.9. Home Performance, Behavior and Adaptation

Through Chi-square tests, cross-tabs were generated for home characteristics and their relationship with the concepts: home performance, behavior, and adaptation. They are presented in Table 3, Table 4 and Table 5, respectively.

Regarding the high dedication to telework/study, 73% lived with two–four cohabitants, making those who lived alone or with one other person, or with more than five people, a minority. In relation to the surface area of the dwelling, this high dedication was distributed, with 43.2% of dwellings being between 62.5 and 145.5 m^2^, even though slightly more than a fifth lived in dwellings of larger surface area, and a third occupied dwellings smaller than 62.5 m^2^. Regarding the type of home, 72.3% lived in houses, while more than a quarter lived in flats.

A great majority of the sample had recreation areas (83.7%). Almost all of those who teleworked obviously had the internet, and therefore did not have to resort to other means to go online.

The perception of lighting in general was adequate for almost all of the highly dedicated teleworkers (90.1%). Regarding air quality, 73.8% of them also found it favorable.

However, noise insulation was adequate for slightly more than half, but not adequate for almost the other half (47.5%).

Regarding temperature, 46.8% of those who teleworked with high dedication declared that they were in comfort, while more than half of the sample were not. Regarding heating, half of the sample used it rarely or only if necessary, while 42.3% did not use it at all. Of the total sample, 8% used it continuously.

This compares with the use of air conditioning that was reported, since for this part of the sample, 64.7% declared only using it rarely or strictly if necessary. A fifth used it more continuously, and 15% did not use it at all.

Regarding statistically significant relationships (*p* value <0.05), dedication to telework was related to the useful surface of the home, so that, the larger the surface, the greater the dedication, and also with the quality of lighting in general (natural as well as artificial). The significance of these relationships can be explained if the context is taken into account: if the majority of the sample has declared to have between three and five members or more in the dwellings, aspects such as isolation from the rest of the household, concentration, and good lighting to carry out tasks in a comfortable and pleasant way, were essential. Of the rest of the home qualities, not having a significant relationship implied that they did not intervene in the temporary dedication to teleworking. This also reveals that living in single-family homes or apartments, having or not having recreational areas, living with more or fewer people, or having a certain indoor environmental quality related to noise, air quality or thermal comfort, did not affect the sample’s time-dedication to teleworking. Therefore, teleworkers organized their schedules according to other priorities or reasons, with no incidence of such home aspects.

Regarding the behavior of the cohabitants, those who changed more than five habits of their day to day stood out, compared to those who changed less than five. Focusing on the results of those who modified the most habits, the sample remains constant in terms of to the number of cohabitants in the home, around three quarters, regardless of whether they changed more or less habits. However, for those with more modified habits, almost half of the sample (46.5%) had an average size of dwelling, with a range of 62.5–145 m^2^. A further 30% lived in houses smaller than 62.5 m^2^, and a quarter lived in houses larger than 145 m^2^. According to the type of dwelling, those who changed more habits, comparatively, tended to live in flats more than those who changed fewer during confinement.

On the availability of recreational spaces, the proportion between households with more or less modified habits always remained around 84%. On the adequate indoor environmental quality, both in the case of natural lighting (89%), air quality (72%), or noise insulation (52%), the sample remained stable regardless of the number of modified habits. Similarly, in terms of comfort, slightly less than half of the sample found comfort, independently of the modified habits, although a decrease of more than half a percentage point was observed in favor of those who have made fewer changes in their day to day. In the case of heating, those who adopted more changes during confinement tended to use it less, whilst air conditioning was more used by those with more alterations in their daily life.

Statistically significant relationships were established for: (1) the useful floor area of the dwelling (the larger the floor area, the more habit changes were adopted); (2) the type of dwelling (those with houses made fewer changes than those who lived in flats); and (3) the availability of the internet, which implied a greater number of altered habits.

Regarding the spatial adaptations that were made in the home, it is observed that these increased according to the number of people in the home. On the other hand, the less useful the area in the house, the fewer the changes carried out in the house. The houses in which the most changes were made had changes in the floors, although it did not mean a significant change with respect to the houses. The availability of recreation areas did not significantly change the spatial adaptation of the home, nor did the availability of the internet; these two variables being aspects that do not affect the interior spatial performance of the home, so it is logical. Regarding indoor environmental quality, of the three related variables (general lighting, air quality and noise insulation), only the first had a real impact, since those who had better lighting made fewer spatial adaptations than those who did not. The insufficient air quality slightly changed the spatial adaptations, while the noise had something more to do with the decision to adapt the spaces, but neither of these two significantly.

Regarding temperature, those with more comfort altered their spatial configuration less than those who were in discomfort, although the relationship between the two did not express significance. Those that made fewer changes used heating more, and air conditioning less. Nevertheless, the use of these systems, both heating and cooling, had a slight impact on spatial adaptation. Therefore, as significant variable, only the perception of general lighting stood out.

The reading that can be made of these results is that this variable, overall lighting, was enough reason for spatial adaptation per se, while the remaining variables, although certain trends were observed, cannot be significantly associated with making this decision. It is striking that the largest houses showed the most spatial adaptations. Perhaps it may be due to the little room for modification offered by the small homes themselves, and therefore there was not much capacity for change. It was expected that households living in flats would show more interest in changing, since normally the dimensions of the spaces were smaller, but these functional or distributional alterations could be undertaken.

### 4.10. Qualitative Analysis

**Figure 15:**
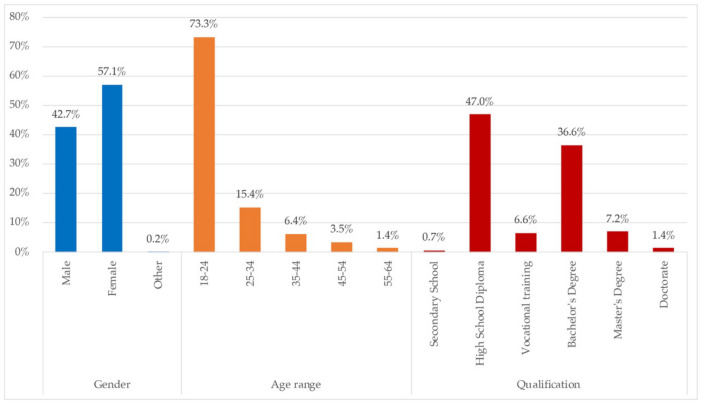
Population distribution of participants who contributed photographs to the mixed questionnaire.

The photographs taken by participants were requested in order to know the environment where they carried out their teleworking and tele-study activities. Figure 15 shows the population sample of the participants who provided photographs. Workspaces in the images were grouped into four spaces: (a) bedrooms, (b) living/dining rooms, (c) studies, and (d) others.

Figure 15 shows the distribution of the qualitative sample. These home representatives answered to the requirement of a telework/tele-study photo, tags, and the questions on those spaces and other reflections on the lockdown and the dwelling. A greater representation of women (57.1%), young people (18–24%), those with at least with a High School Diploma (47%) or who had even completed university studies (45.2%) define the qualitative participants.

### 4.11. Categorization of Images

The teleworking and tele-study spaces were categorized into four spaces: (a) bedrooms, (b) rooms/dining rooms, (c) studies, and (d) others.

(a) Bedrooms

Photos in this category showed that teleworking displaced the leisure activities that were carried out in the bedrooms, obstructing circulation and disabling doors. In some cases, desks were adapted, but, in others, people had to work and study directly on their beds.

(b) Living/Dining rooms

For their part, when social areas such as living and dinning rooms hosted teleworking and tele-study activities, they had to be even more flexible, as they were the spaces that families used several times a day to gather for lunch and dinner, or share free time. Therefore, students and workers had to utilize working mobile stations, creating transit spaces, when possible.

(c) Studies

The studies, as telework spaces, on the contrary, had the capacity to maintain and contain telework activities. However, a small percentage of the surveyed dwellings had these predetermined and equipped spaces to receive study activities and remote work.

(d) Others

Finally, the last category, where users adapted other kind of spaces, different from the previous ones, to carry out work and study activities remotely. This solution of occupying those spaces was prioritized to solve or cover some workspace aspects, sacrifizing others: space, lighting, furniture, connectivity, among others. Additionally, decisions from the teleworkers that chose these spaces, had to be made to respond to certain causes, such as housing size and household composition, internet connexion, need to be isolated for concentration, or contact with outdoor spaces and daylight, for instance. However, clearly this decisions had to be made at the expense of other aspects such as ergonomics, or the overall adequacy of the telework space.

### 4.12. Word Frequencies and Clouds

Table 6 shows the frequencies and word clouds that were obtained by participants.

### 4.13. Most Relevant Verbatims

In Table 7, the 5 most relevant testimonies were selected from both the explanatory responses to the image made, as well as from the responses of reflections and suggestions related to housing and confinement.

## 5. Analysis of Results

This study has representation in 24 of the 32 states of the Mexican Republic, with the largest group of participants being in Mexico City, with 489 surveys answered, equivalent to 50.4% of the sample, followed by the State of Mexico, with 378 surveys and 39% representativeness.

On the characteristics of the homes observed, it is not surprising that Figure 2 states that the largest number of homes surveyed, 35.30%, had an urban environment, and that only 11.80% reflected the situation of rural housing. It is noted that the predominant data were single-family homes in urban environments, mostly (72.81%) houses between 62.5–97.5 m^2^ (22.82%), followed by houses of 145 m^2^ or more. (22.05%) where three to five inhabitants coexist (73.60%). In these households, people who indicated open areas inside the houses had patios (50.20%). This percentage was significantly reduced by reporting only 32.90% of the existence of gardens or green areas inside the house.

With regard to the habitability characteristics related to lighting and air quality, people perceived that those were adequate or very adequate, so it follows that there must have been enough windows to allow the house to be ventilated and illuminated naturally. Although 41.22% indicated that noise insulation inside the home was adequate, a significant percentage, 37.07%, felt that housing was not insulated from noise, which also suggest that such dwellings could have simple insulation, a constructive solution that is mostly used in Mexican housing, rather than in another architectural typology, due to costs. It was also observed that in the place where there were most commonly windows within the sample were the bedrooms, with 82% frequency, followed by living rooms (75.05%) and kitchens (69.18%). Surprisingly, despite the regulations, 100% registration of the existence of windows was not reached, which is a determining factor of physical and mental health.

On the comfort and use of heating inside the house, most of the houses (77.04%), reported not having any active air conditioning. These data are consistent, because the most predominant sample came from Mexico City and the State of Mexico, cities that, because of their geographical location and level of urbanization, have a mild climate, which initially does not require the implementation of heaters (Figure 6) or cooling systems (Figure 7). However, more than a half of the sample declared not feeling comfortable in their homes.

On the other hand, regarding the equipment and supplies of the house, the inhabitants report that 62.24% that their consumption of domestic hot water has not changed during confinement, and that the appliances that had had more use, compared to the usual, were mobile devices such as mobile phones and tablets, as well as their personal and laptop computers. Household cleaning habits have been carried out according to the same routine in the homes surveyed, and this is reflected in the use of household appliances related to this activity, such as the dryer and washing machine, the vacuum, dishwasher and cleaning robots, which had the same use as that prior to confinement. Thus, it was also reported that there were no changes in general in the use of appliances related to food storage and production, where the oven, stove and freezers had a normal activity, contrary to what was expected by the closure of restaurants. Nevertheless, at least 20% of participants declared to have increased each of the most common appliances during confinement.

In relation to energy consumption patterns, respondents mostly perceived (45.19%) to have increased their energy consumption. For its part, the strategy of turning off the lights (57.02%) as well as turning off equipment that was not in use (32.20%) were the most recurrent.

Finally, with regard to the changes they would make to their home if they could, it is emphasized that people were happy with the size of their home, since only 7.42% indicated that they would change it. What 32.68% of the occupants referred to was that they would like was first and foremost to have a space for plants, pots or directly a garden. This answer went hand in hand with the fact that people would change the views outside their homes if they could.

According to Section 4.9, where possible relations among home characteristics and main aspects in this research (performance, behavior and adaptation) were held, the results highlighted two relevant findings. The first one, that certain home features were statistically related to these aspects, such as dwelling floor area, and general lighting, and also, for behavior, dwelling type and internet availability. As mentioned, these relations could respond to priorities in workspace characteristics, such as daylight, sacrificing other aspects, such as ergonomics. Other relations could be explained from a certain status or socio-economic perspective, for example having a bigger house, and therefore being able to make more changes and spatial adaptations indoors. Additionally, linked to this status, people who lived in better houses, with faster internet connection, had a variety of habits (for instance, social networks, leisure, or sports), more likely to be affected by confinement, than others whose lives were reduced to work, family care and home.

With regard to qualitative analysis, the spaces for telework or tele-study were categorized into four groups, in which the degree of adaptation of the participants can be observed using a scale from exclusive spaces to shared spaces. Some images show how places that were initially not intended for work or study were used, such as bedrooms, living rooms or dining rooms. In those spaces, the furniture was not intended for teleworking, so people had to resort to spatial or functional adaptations, sacrificing ergonomics, for instance. It is especially relevant when photos showed the use of beds and dining-room furniture for remote work or study. As mentioned in Section 4.9., the relevance of lighting, and especially daylight, could make teleworkers move inside the house, seeking better-lit areas (also related to window availability, which was more present in social and main spaces, such as living/dining rooms, and bedrooms, respectively). Bearing in mind other preferences, such as internet connectivity, isolation from other cohabitants, enough room to spread their notes and technological devices, it mainly depended on the household circumstances and house characteristics, as also showed the verbatims included in Table 7. In the analysis of the texts through their frequency, the importance of space was emphasized again, with this term appearing in all clouds, followed by the importance of daylight and artificial lighting, in addition to the concept “comfortable”. Finally, in the analysis of verbatims, several significant examples are shown, highlighting the importance of having sufficient space, the need to share it, or have adequate means (internet connection, good lighting quality, etc.), among others. In addition, time in confinement reflected on both personal and collective improvement, as well as underlining the value of housing.

## 6. Discussion

The main public health measure adopted by most countries, including Mexico, was confinement. The home became the main refuge for all people. Many of them were forced, or at least encouraged, to cohabit and to spend as much time as possible in their homes. In the case of Mexico, the government did not force quarantine, and just recommended that citizens stay home voluntarily, especially those with breathing problems [64]. In the study, it stands out that most participants lived in company. This led to sharing spaces and tasks among the cohabitants. Spending more time at home has meant greater exposure to the conditions of habitability, indoor environmental quality, and comfort at home. People’s shortcomings and preferences were interpreted as conditions of adaptability of their housing to new requirements due to the health emergency. This study has dealt with the evaluation of housing adaptation-capacity (resilience), bearing in mind the multiple tasks and how they were satisfied to achieve quality in people’s lives: space distribution and adaptation; behavior patterns and changes; ventilation and lighting habits; indoor environmental quality; thermal supplies, facilities and appliances availability and use frequency; and perceived comfort.

Resilience has been addressed from different perspectives and is also an ever-evolving concept. From a household point of view, it relates to multiple factors, including those linked to household income and assets. Specifically, resilience refers to “the ability of households to cope with basic needs of livelihood in case of emergency or shock” [65]. In more detail, resilience in social housing must contain four fundamental aspects from the point of view of the household that inhabits it, throughout its useful life cycle: habitability, sustainability, accessibility and flexibility [66].

While participants reported adequate lighting and air quality, a little over a third expressed that their homes were not adequately insulated from noise. A study in 70 Mexican cities indicated that the confinement situation led to an increase in the number of conflicts between neighbors, with 42% related to noise [67]. Considering that noise pollution is an environmental variable that relates to the incidence and severity of COVID-19 [68], we must take into account the architectural importance of insulation in the design and rehabilitation of homes.

In relation to the change in personal and work habits, they highlighted the increase in the use of electronic means. Nearly half saw higher energy consumption during confinement. A study in New York found that in March 2020 there was a 23% increase in household consumption. Some energy experts recommended an awareness campaign during the confinement of users in the conservation of the energy of buildings, promoting a more responsible change in behavior [69]. Undoubtedly, in addition to the health impact, the pandemic has had a strong impact on energy consumption at all levels, so it would be time to initiate a transformation of renewable or green energy sources [70].

According to the home performance, behavior and adaptation, the interpretation of results could be discussed, due to several reasons: firstly, the statistical relations showed that the main home characteristics that intervened in these three concepts were mainly the dwelling floor area, the lighting, and, for the case of behavior, the housing type and the internet availability, that could be also related to a higher status and socio-economic condition. A reflection was exposed previously, on the idea that people with higher socio-economic status and resources were more likely to adapt their dwellings to new requirements; at the same time, this status allowed them to have greater social capital and networks, and, therefore, when confined, they had to abandon or adapt more activities and spaces to the new circumstance. The essential or blue-collar workers, were not able to be locked down, either the impossibility to adapt their tasks to remote work, or to avoid losing their works or incomes [71]. So, they probably did not carry out so many adaptations, or habit changes in their lives, compared with people who were allowed to telework or tele-study. Moreover, since in Mexico the quarantine was not mandatory, this could why the study did not perceive more significant relationships between the home characteristics and the home performance, behavior and adaptation. It was probably more a question related to the awareness of households, and their interpretation of the health emergency and the need to be protected and to practice social distancing.

The use of photos, tags and narratives helped to understand and enrich the information obtained through the close-ended questions, since insights merged from this kind of qualitative data. The use of qualitative research alone or in mixed-methods help researchers to understand complex phenomena, either adding, nuancing or explaining the circumstances from another perspective and approach. Since photos are graphical, they give visual information of private areas, relevant for the researchers who could not access to this level of information in any other way.

According to the results of the qualitative analysis, the need to rethink a new design has been highlighted to enable spaces that allow more than one activity or function, including telework or tele-study. Therefore, in the post-COVID-19 era, the idea of flexible housing is increasingly attractive, and even more so, the preference of the population will be to live in larger, better-lit places and with pleasant views outside, as shown Figure 14, which were the most valued characteristics surveyed. Moreover, when contrasting this information with Figure 5, concerning windowed spaces, it stood out that 86.68% of the homes surveyed had a window per bedroom, which, although it was a high percentage, is not high enough, as building regulations require 100%. This shows that some buildings remain outside the regulations [72].

On the other hand, occupant density in buildings conditioned energy consumption, and posed a direct link between COVID-19 transmission risk and the number of occupants [73]. This should lead us to reflect on teleworking as a good preventive or public health containment measure, to the extent that the scope and employment situation permit.

In addition, in the case of Mexico, in particular Mexico City, it is estimated that transportation spending can account for one third of a minimum-wage worker’s salary. Therefore, teleworking could be an option as a saving measure for workers [74] in terms of incomes and time on journeys [75], which facilitates a higher quality of life [76]. This would entail a reflection on teleworking as a good preventive or public health containment measure, to the extent that the scope and employment situation allows it.

Another consideration about teleworking has been the positive impact on air quality in cities, due to reduced emissions, mainly due to vehicle use restrictions caused by the confinement of people in households [77]. This fact is an added value in the integration of housing and telework; an issue that should be a key element in the future design of households. This, combined with the reduction of fossil fuels in the country, can lead to comprehensive models for the indiscriminate reduction in mobility for sustainable development.

However, the lack of other supplies, such as internet connectivity, has also been highlighted, bearing in mind that in many households, inhabitants have had to share the connection. Where home connectivity is not possible, it is necessary to rethink the possibility of designing and implementing shared workspaces as a possible solution [74].

The study has several limitations, specifically affecting the quantitative approach. The main one comes from the sampling strategy itself. A convenience sampling never represents the whole universe, because it is not randomized. The second one, due to the nature of the recruitment channel used, online, limited the participation only to those who had digital resources and internet connection, another reason to not be able to generalize the findings provided. Recent studies indicate that 70% of the Mexican population has access to the internet [78]. Likewise, it should be noted that a significant number of Mexican families (6 out of 10) live in rural areas where, in addition to economic precariousness, digital infrastructures are still very deficient compared to digital infrastructures in urban areas [79].

Another limitation is related to the term “resilience” in a broader definition. In this study, the term is contextualized to the confinement phenomenon, as a measure adopted by countries, suggested for Mexico in this case, to protect people from COVID-19. Other emergencies, such as natural disasters, are not included in the study scope, and require a detailed analysis from the perspective of housing design, particular population characteristics and needs, valuating specific risks and consequences.

## 7. Conclusions

The house can be understood as a complex system, capable of mutating its functions in an emerging way beyond itself, to become offices and schools in a space designed for daily activities and leisure, hence its adaptive capacity is reported in terms of resilience. While the adaptations of the house were not in its physical infrastructure, they were based on the new functions it received. Some traditional functions had to be segregated to make room for new ones, such as dining rooms and bedrooms, whose usual functions of eating and rest, were replaced in whole or in part by telework or tele-study.

The findings suggest that people felt consciously satisfied with the housing characteristics in general. Overall lighting and air quality seemed to be suitable for most of them. The aspects that did not reach satisfactory levels for a significative part of the sampling were mainly the noise insulation (48%), and thermal comfort (52%). It was not common for the most of the surveyed homes to have heating and cooling systems to meet and maintain comfort indoors. It stood out the fact that 63% of surveyed homes had windows in the main rooms, whilst a maximum of 82% had it in bedrooms. Therefore, it is more than likely that a large part of Mexican homes were not ventilated properly, although many of them had patios, gardens, or other open spaces in contact with the outside.

All of the spatial changes related to the hygiene and safety measures were followed and adopted in the homes, and daily households’ habits were adjusted to the new circumstances. The sample was mainly devoted to telework or tele-study, care and domestic chores, and spaces dedicated to telework were bedrooms, but also other shared and improvised spaces, with the subsequent adaptation to the furniture availability, and using laptops, thus forgetting concepts such as ergonomics.

According to these findings, it is urgent to undertake retrofit measures to ensure adequate housing to the minimum requirements of habitability, comfort and safety, with clear and proven implications on health and well-being. Self-construction is an extended practice, out of the regulation scope, and thus with no guarantees for safe and healthy living. Beyond government plans and strategies to build and retrofit the residential park, other population measures are needed to stop this practice. Affordable, sustainable and habitable housing as a human right, is necessary, even when it is presented as the only indoor safe space. If so, as governments have presented in their “Stay home” campaigns, boosting telework and tele-study for more people’s protection, it has to be guaranteed in all aspects in the home.

This study contributes to laying the foundations regarding the adequacy of Mexican homes in the face of confinement, in terms of habitability, resilience and general satisfaction of the homes. However, given its exploratory nature, it is necessary to delve into these aspects with a randomized and a greater scale study, to have a representative knowledge of the Mexican population. In turn, other minority populations, such as indigenous or vulnerable populations in general, could be the object of detailed analysis through participatory qualitative, ethnographic or other studies, which help to understand what it means for their day-to-day life and for their situation of vulnerability to adopt a measure such as confinement. These could be interesting future lines of research, which would also help to assess the present and future implications that this measure may entail for the population.

One solution would be to design contingency plans for these types of scenarios, bearing cross-cutting benefits to mitigate the consequences of confinement, such as increased energy consumption, overexposure to unhealthy or inadequate domestic spaces, and the need to find the right home comfort for longer hours than usual. Some of the measures that could bring about a greater social, economic and health impact on these population segments could be: rehabilitating homes or facilitating access to other more habitable, spacious or healthy ones; raising public awareness through citizen participation and empowerment in housing, health and well-being; undertaking population training in matters of health and habitability in housing, and discouraging practices such as self-construction; and planning strategies and incentives for the energy and ecological transition linked to the use of certain fuels in homes, and their impact on health.

Whilst there are many studies that address homes in relation to health, few delve into the domestic space. In this form, this research has helped by addressing a gap in knowledge and understanding of the affectations that the most important pandemic in modern times has had in the built spaces, contributing significantly to the field of housing and habitability during prolonged confinements. It has been seen that the housing model of the future should contemplate adaptive forms. The architectural program of housing in this digital era has been questioned, which does not include spaces or services that allow it to comply with remote activities. Paradoxically, it has become clear that the other lacking elements reported by the inhabitants go hand-in-hand with the connection with the natural environment that urbanization has marginalized.

This study contributes to filling the gap in knowledge of the theoretical and comprehensive framework on housing. For this, the mixed method was carried out, a multiple approach often applied in the theoretical development of the house.

Government-level strategies to promote and favor access to more sustainable, safe, habitable and resilient housing in the face of such extreme measures suggest that efforts should be made by all the agents involved: political decision-makers, administrations and institutions, architects and urban planners, as well as public–private cooperation, in order to guarantee both prevention and relief initiatives in the face of this type of contingency.

Finally, since isolation is undeniably useful in reducing the spread of communicable diseases, it is not entirely useful for safeguarding people’s physical and mental health. That is the reason why different authors point out the importance of new housing conditions and the design of surrounding areas as well as urban design in general, such as safe public spaces for exercise and walking to deal with situations that, as with the current pandemic, require new scenarios of confinement or physical distancing.

This is the reason why the contributions of this article, with respect to the deficiencies and preferences of users about their homes, can be taken as a starting point for the updating of the Mexican Government’s National Strategic Development Plans. Those, aligned with the Sustainable Development Goals from the UN, will contribute to the sustainable development of the country’s housing and infrastructure in the future.

## Figures and Tables

**Table 1:** Quantified categories and variables from the questionnaire.

Categories	Variables
Sociodemographic profile of participants	Gender, age, highest level of studies completed, habitat.
General housing taxonomy	Number of people in the household; useful room area of the house; type of housing; available recreation areas; internet availability.
Behavioral patterns (use and occupation)	Temporary dedication to tasks; modified habits; house spatial adaptation.
Facilities and appliances	Appliances and comparative use *; Digital equipment and comparative use; Domestic Hot Water, power source and comparative use.
Energy expenditures and saving strategies	Estimated alteration of energy bills; energy saving strategies.
Indoor environmental quality	Overall lighting; air quality; noise insulation; windowed housing spaces.
Comfort	Thermal sensation (declared comfort); heating and cooling systems, types and use in confinement.
Desired housing improvements	Preferences for housing improvements.

* Comparative use is referred to pre-confinement and confined periods.

**Table 2:** Descriptive statistics summary of the sample.

	N	Mean	Max	Min	S.D.
Gender	958	1.5996	3	1	0.49446
Age range	962	1.4482	6	1	0.92457
Qualification	956	5.0332	8	2	1.14488

**Table 3:** Relationships among home features and performance.

Performance:		Temporary Dedication to Telework/Study			
Variable	Total N (% Column)	1–2 (% Column)	3 (% Column)	4–5 (% Column)	*p* *
**General**	925 (100)	64 (6.9)	181 (19.6)	680 (73.5)	
**Number of people in the household**					0.636
1–2	124 (13.7)	11 (17.7)	21 (11.9)	92 (13.8)	
3–5	666 (73.5)	42 (67.7)	136 (77.3)	488 (73.1)	
>6	116 (12.6)	9 (14.5)	19 (10.8)	88 (13.2)	
**Useful room area of the house**					0.014
30–62.5 m^2^	326 (36.1)	34 (55.7)	67 (38.5)	225 (33.6)	
62.5–145.5 m^2^	378 (41.8)	18 (29.5)	71 (40.8)	289 (43.2)	
>145 m^2^	200 (22.1)	9 (14.8)	174 (20.7)	155 (23.2)	
**Type of housing**					0.733
Flat	248 (27.3)	18 (29)	44 (25)	186 (27.7)	
House	661 (72.7)	44 (71)	132 (75)	485 (72.3)	
**Available recreation areas**					0.680
No	156 (16.9)	13 (20.3)	32 (17.7)	111 (16.3)	
Yes	769 (83.1)	51 (79.7)	149 (82.3)	569 (83.7)	
**Internet availability**					0.810
No	40 (4.6)	3 (5.3)	9 (5.5)	28 (4.3)	
Yes	826 (95.4)	54 (94.7)	156 (94.5)	616 (95.7)	
**Overall lighting**					0.000
Not suitable	93 (10.9)	15 (27.3)	15 (9.3)	63 (9.9)	
Suitable	760 (89.1)	40 (72.7)	147 (90.7)	573 (90.1)	
**Air quality**					0.095
Not suitable	240 (28)	21 (37.5)	51 (31.9)	168 (26.2)	
Suitable	617 (72)	35 (62.5)	109 (681.1)	473 (73.8)	
**Noise insulation**					0.700
Not suitable	417 (48.3)	30 (52.6)	81 (49.7)	306 (47.5)	
Suitable	447 (51.7)	27 (47.4)	82 (50.3)	338 (52.5)	
**Thermal sensation**					0.236
Discomfort	445 (52.2)	31 (57.4)	76 (46.6)	338 (52.2)	
Comfort	407 (47.8)	23 (42.6)	87 (53.4)	297 (46.8)	
**Use of heating in confinement**					0.577
Never	83 (43)	4 (44.4)	16 (45.7)	63 (42.3)	
Rarely-Strictly	90 (46.6)	4 (44.4)	13 (37.1)	73 (49)	
Quite-Continually	20 (10.4)	1 (11.1)	6 (17.1)	13 (8.7)	
**Use of air aconditioning in confinement**					0.938
Never	55 (14.3)	4 (12.9)	10 (13.2)	41 (14.7)	
Rarely-Strictly necessary	247 (64.2)	20 (64.5)	47 (61.8)	180 (64.7)	
Quite-Continually	83 (21.6)	7 (22.6)	19 (25)	57 (20.5)	

* *p* value for the chi-square test of the variable’s relationship with performance. A *p* < 0.05 implies a statistically significant relationship.

**Table 4:** Relationships between home features and behavior.

Behavior:		Modified Habits during Lockdown		
Variable	Total N (% Column)	1–4 (% Column)	5–14 (% Column)	*p* *
**General**	970 (100)	464 (47.8)	506 (52.2)	
**Number of people in the household**				0.079
1–2	124 (13.6)	44 (10.9)	80 (15.9)	
3–5	669 (73.6)	305 (75.3)	364 (72.2)	
>6	116 (12.8)	56 (13.8)	60 (11.9)	
**Useful room area of the house**				0.000
30–62.5 m^2^	328 (36.2)	182 (45)	146 (29)	
62.5–145.5 m^2^	379 (41.8)	145 (35.9)	234 (46.5)	
>145 m^2^	200 (22.1)	77 (19.1)	123 (24.5)	
**Type of housing**				0.000
Flat	248 (27.2)	86 (21.2)	162 (32)	
House	664 (72.8)	320 (78.8)	344 (68)	
**Available recreation areas**				0.646
No	156 (16.1)	72 (15.5)	84 (16.6)	
Yes	814 (83.9)	392 (84.5)	422 (83.4)	
**Internet availability**				0.000
No	40 (4.6)	30 (8.3)	10 (2)	
Yes	828 (95.4)	333 (91.7)	495 (98)	
**Overall lighting**				0.810
Not suitable	94 (11)	40 (11.3)	54 (10.8)	
Suitable	761 (89)	314 (88.7)	447 (89.2)	
**Air quality**				0.494
Not suitable	241 (28.1)	96 (26.8)	145 (28.9)	
Suitable	618 (71.9)	262 (73.2)	356 (71.1)	
**Noise insulation**				0.552
Not suitable	417 (48.2)	170 (47)	247 (49)	
Suitable	449 (51.8)	192 (53)	257 (51)	
**Thermal sensation**				0.102
Discomfort	446 (52.2)	170 (48.9)	276 (54.5)	
Comfort	408 (47.8)	178 (51.1)	230 (45.5)	
**Use of heating in confinement**				0.407
Never	83 (42.8)	36 (39.6)	47 (45.6)	
Rarely-Strictly necessary	91 (46.9)	43 (47.3)	48 (46.6)	
Quite-Continually	20 (10.3)	12 (13.2)	8 (7.8)	
**Use of cooling in confinement**				0.124
Never	56 (14.1)	29 (16.6)	27 (12.2)	
Rarely-Strictly necessary	256 (64.5)	116 (66.3)	140 (63.1)	
Quite-Continually	85 (21.4)	30 (17.1)	55 (24.8)	

* *p* value for the chi-square test of the variable’s relationship with behavior. A *p* < 0.05 implies a statistically significant relationship.

**Table 5:** Potential relationships among home features and adaptation.

Adaptation:		House Spatial Adaptation		
Variable	Total N (% Column)	1–3 (% Column)	4–6 (% Column)	*p* *
**General**	791 (100)	731 (92.4)	60 (7.6)	
**Number of people in the household**				0.932
1–2	110 (14)	102 (14)	8 (13.3)	
3–5	572 (72.6)	529 (72.7)	43 (71.7)	
>6	106 (13.5)	97 (13.3)	9 (15)	
**Useful room area of the house**				0.101
30–62.5 m^2^	282(35.8)	266 (36.5)	16 (26.7)	
62.5–145.5 m^2^	336 (42.6)	311 (42.7)	25 (41.7)	
>145 m^2^	170 (21.6)	151 (20.7)	19 (31.7)	
**Type of housing**				0.925
Flat	220 (27.8)	203 (27.8)	17 (28.3)	
House	571 (72.2)	528 (72.2)	43 (71.7)	
**Available recreation areas**				0.500
No	130 (16.4)	122 (16.7)	8 (13.3)	
Yes	661 (83.6)	731 (83.3)	60 (86.7)	
**Internet availability**				0.263
No	36 (4.6)	35 (4.8)	1 (1.7)	
Yes	753 (95.4)	694 (95.2)	59 (98.3)	
**Overall lighting**				0.007
Not suitable	87 (11.2)	74 (10.3)	13 (21.7)	
Suitable	691 (88.8)	644 (89.8)	47 (78.3)	
**Air quality**				0.481
Not suitable	217 (27.7)	198 (27.4)	19 (31.7)	
Suitable	565 (72.3)	524 (72.6)	41 (68.3)	
**Noise insulation**				0.404
Not suitable	380 (48.2)	348 (47.7)	32 (53.3)	
Suitable	409 (51.8)	381 (52.3)	28 (46.7)	
**Thermal sensation**				0.345
Discomfort	413 (52.5)	378 (52)	35 (58.3)	
Comfort	374 (47.5)	349 (48)	25 (41.7)	
**Use of heating in confinement**				0.907
Never	79 (43.9)	73 (43.5)	6 (50)	
Rarely-Strictly	84 (46.7)	79 (47)	5 (41.7)	
Quite-Continually	17 (9.4)	16 (9.5)	1 (8.3)	
**Use of air conditioning in confinement**				0.148
Never	48 (14)	43 (14)	5 (14.7)	
Rarely-Strictly	217 (63.5)	200 (64.9)	17 (50)	
Quite-Continually	77 (22.5)	308 (21.1)	12 (35.3)	

* *p* value for the chi-square test of the variable’s relationship with adaptation. A *p* < 0.05 implies a statistically significant relationship.

**Table 6:** Frequency and word clouds of total tags and descriptive responses and reflections or suggestions of photos made about the participants’ workspace or study.

Word Clouds	Words	Frequency	Percentage
Photo labels			
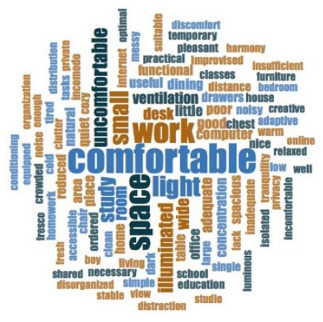	comfortable	130	6.87%
	space	100	5.29%
	work	99	5.23%
	light	73	3.86%
	small	66	3.49%
	study	50	2.64%
	uncomfortable	46	2.43%
	illuminated	44	2.33%
	room	29	1.53%
	wide	29	1.53%
	desk	26	1.37%
Description and reason to take photo			
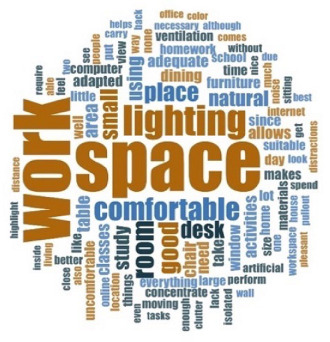	space	211	6.86%
	work	201	6.54%
	lighting	97	3.15%
	comfortable	66	2.15%
	room	60	1.95%
	good	55	1.79%
	place	51	1.66%
	small	45	1.46%
	desk	45	1.46%
Thought or suggestion related to home and lockdown			
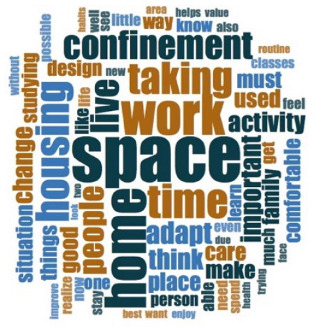	space	117	2.76%
	home	88	2.08%
	work	86	2.03%
	time	71	1.67%
	housing	70	1.65%
	taking	66	1.56%
	confinement	59	1.39%
	live	59	1.39%
	people	49	1.16%

**Table 7:** Selection of verbatim records from answers about the photographs and personal reflections and suggestions.

Verbatim Records
Explanation of the image made
*“The location of the nearby internet modem for the signal, Good background for video calls. The location of the desk is fine by the lighting so it doesn’t look dark”*
*“It is a very small space, it is in the middle of the living room and the dining room without the possibility of moving it elsewhere due to the lack of space”*
*“It is a temporary desk that is located in the dining room of my house; this is because also my two sisters’ study”.*
*“The natural light that enters through the window is very good and the plants help to have that harmonious atmosphere”.*
*“I would emphasize that I put a pillow in the chair because I get tired in the back after sitting for long periods of time”.*
Reflections and personal suggestions
*“You have to take advantage of every moment, from the most basic to the biggest”.*
*“It is hard to adapt to a new way of working, but you can always be flexible”.*
*“That it is something that I really don’t want to repeat, but it has taught me to know myself”.*
*“Make spaces more suitable for greater comfort in our own homes”.*
*“Realize how important it is to have a space to relax, a green area or a break room”.*

## Data Availability

The data are not publicly available due to ethical reasons.
